# TNF Accelerates Death of Mandibular Condyle Chondrocytes in Rats with Biomechanical Stimulation-Induced Temporomandibular Joint Disease

**DOI:** 10.1371/journal.pone.0141774

**Published:** 2015-11-03

**Authors:** Hongxu Yang, Mian Zhang, Xin Wang, Hongyun Zhang, Jing Zhang, Lei Jing, Lifan Liao, Meiqing Wang

**Affiliations:** State Key Laboratory of Military Stomatology, Department of Oral Anatomy and Physiology and TMD, School of Stomatology, the Fourth Military Medical University, 145 Changle West Road, Xi’an, China; University of Umea, SWEDEN

## Abstract

**Objective:**

To determine if temporomandibular joint chondrocyte apoptosis is induced in rats with dental biomechanical stimulation and what a role TNF takes.

**Methods:**

Thirty-two rats were divided into 4 groups (n = 8/group) and exposed to incisor mal-occlusion induced by unilateral anterior crossbite biomechanical stimulation. Two groups were sampled at 2 or 4 weeks. The other two groups were treated with local injections of a TNF inhibitor or PBS into the temporomandibular joints area at 2 weeks and then sampled at 4 weeks. Twenty-four rats either served as unilateral anterior crossbite mock operation controls (n = 8/group) with sampling at 2 or 4 weeks or received a local injection of the TNF inhibitor at 2 weeks with sampling at 4 weeks. Chondrocytes were isolated from the temporomandibular joints of 6 additional rats and treated with TNF *in vitro*. Joint samples were assessed using Hematoxylin&eosin, Safranin O, TUNEL and immunohistochemistry staining, real-time PCR, fluorogenic activity assays and Western blot analyses. The isolated chondrocytes were also analyzed by flow cytometry.

**Results:**

Unilateral anterior crossbite stimulation led to temporomandibular joint cartilage degradation, associated with an increase in TUNEL-positive chondrocytes number, caspase-9 expression levels, and the release of cytochrome c from mitochondria at 2 weeks without changes in TNF and caspase-8 levels until after 4 weeks. TNF stimulated apoptosis of the isolated chondrocytes and up-regulated caspase-8 expression, but did not change caspase-9 expression levels. Local injection of TNF inhibitor down-regulated caspase-8 expression and reduced TUNEL-positive cell number, but did not reverse cartilage thickness reduction, caspase-9 up-regulation or cytochrome c release.

**Conclusions:**

Unilateral anterior crossbite stimulation induces mitochondrion-mediated apoptosis of articular chondrocytes. TNF accelerated the unilateral anterior crossbite induced chondrocytes apoptosis via death-receptor pathway. However, anti-TNF therapy does not prevent cartilage loss in this model of temporomandibular joint.

## Introduction

Biomechanical factors play important roles in several forms of osteoarthritis (OA), including temporomandibular joint disorder (TMD) [1]. Based on biomechanical principles [2], we created an abnormal dental occlusion, an experimentally created type of disordered occlusion that induces OA-like changes in the local cartilage of the temporomandibular joint (TMJ) [3–7] accompanied by an increase in articular chondrocyte apoptosis. Recently, we enhanced this form of aberrant dental biomechanical stimulation, which we termed the unilateral anterior crossbite (UAC) model, by attaching metal tube crowns to the left incisor of rats. This greatly accelerated the development and increased the severity of TMJ cartilage degradation [8].

One of the main pathological changes in cartilage in OA is enhanced chondrocyte apoptosis [9, 10] or apoptosis-like programmed cell death [11, 12], which is mediated by two distinct pathways: the mitochondrial and the death receptor pathway [13, 14, 15]. Caspase-9 is activated by mitochondrial pro-apoptotic signaling [16], whereas caspase-8 is activated during tumor necrosis factor (TNF) receptor-mediated apoptosis [17]. However, it is not known if there is increased chondrocyte apoptosis in UAC-induced TMJ degradation or whether and what a role of TNF takes.

The initial morphological characteristics of the mitochondria-dependent or intrinsic apoptotic pathway include condensation of chromatin and the swelling of mitochondria, followed by the release of cytochrome c [18]. In the presence of ATP or dATP, cytochrome c binds to the adaptor protein, apoptotic protease activating factor-1 (Apaf-1), which then recruits pro-caspase-9 to form an apoptosome. Pro-caspase-9 is then autolytically activated to active caspase-9 [19, 20].

Pro-inflammatory cytokines, most notably TNF play a pivotal role in apoptosis, inflammation and tissue damage [21, 22, 23]. However, the function and mechanisms of TNF in OA are inconsistent. For example, some studies have indicated that TNF causes apoptosis [24–27] by binding to the ‘‘death receptor” TNF-receptor-1 (TNF-R1) [17, 28, 29]. This extrinsic apoptotic pathway [27, 30] involves ligand binding to the “death receptor”, followed by transmission of signals to the interior of the cell through Fas-associated death domain protein (FADD) and poly ADP-ribose polymerase (PARP), and finally recruitment of initiator caspases, such as caspase-8, which induce apoptosis [31, 32]. However, other studies have reported that TNF activates anti-apoptotic family proteins, such as bcl-2, without promoting apoptosis [33, 34, 35]. TNF has also been reported to protect against apoptosis [36], maintaining the renewal of local inflammatory mediators by promoting increased expression of cytokines in chondrocytes [37, 38].

In the present study, we investigated if apoptosis occurs in UAC-induced TMJ cartilage and if TNF is involved.

## Materials and Methods

### Experimental animals and grouping

Sixty-two 6-week-old, female, Sprague-Dawley (SD) rats (weight 140–160 g) were provided by the animal center of the Fourth Military Medical University in Xi’an, China. The animal care and all procedures were performed according to institutional guidelines and were approved by the Ethics Committee of the Fourth Military Medical University. All surgeries were performed under sodium pentobarbital anesthesia, and all efforts were made to minimize suffering. Fifty-six rats were equally divided into seven groups and time-points. In the 2-week Control and 4-week Control groups, a mock operation was conducted on the rat incisors, and the animals were sacrificed either 2 or 4 weeks later, respectively. In the 2-week UAC and 4-week UAC groups, unilateral anterior crossbite (UAC) was induced in the left incisors as described below, and the animals were sacrificed either 2 or 4 weeks later. In the UAC+Inhibitor, Control+Inhibitor, and UAC+PBS groups, a TNF monoclonal antibody (see below) or PBS was injected locally into the TMJ area after 2 weeks. The TMJs were sampled after 4 weeks. The mandibular condylar chondrocytes of the other six rats were isolated and treated with TNF in vitro.

### Creation of the unilateral anterior crossbite model

The unilateral anterior crossbite (UAC) model was created following our previous reports [7, 8]. Briefly, the animals were anesthetized with 1% pentobarbital sodium (40 mg/kg). In the experimental groups, a prosthesis (length = 2.5 mm, inside diameter = 3 mm) cut from a 25-guage needle (Shinva Ande, Shandong, China) was glued to the left maxillary incisor using zinc phosphate cement, and a 4.5 mm long, 2.5 mm diameter segment of a 20-guage needle was glued to the left mandibular incisor. The end of the mandibular prosthesis was bent to a 135-degree angle to guide it forward, thus creating a UAC relation of the left side incisors. In the sham control groups, all operations were performed, but the UAC prostheses were removed before the glue hardened. The UAC operation required no more than 3 minutes. No loosening of the prostheses occurred throughout the study period. Rats were carefully fed the same standardized diet throughout the experiment.

### Temporomandibular joint local area injection

Two weeks after UAC stimulation, rats were laid sidelong after induction of deep anesthesia with intraperitoneal sodium pentobarbital. Fifty μl (10 μg/ml) of an anti-TNF monoclonal antibody, TN3-19.12 (Abcam, ab10863, Cambridge, United Kingdom) without sodium azide was diluted in PBS and injected locally into right and left TMJ areas of the UAC+Inhibitor and Control+Inhibitor groups. The same volume of PBS was similarly injected bilaterally into the TMJ areas of rats in the UAC+PBS group. The injections were performed once every two days for 2 weeks [39] until the end of 4-week experimental period. The technique of the injection followed what previously reported [40, 41]. The needle of a specially made Hamilton-type syringe was inserted just below the zygomatic arch between the eye and ear until the outer surface of the mandibular ramus was reached. The orientation of the needle head was adjusted to allow it to go along the bone wall and finally reach the TMJ region.

### Tissue sampling and preparation

All rats were sacrificed by pentobarbital overdose, and the TMJs were sampled after 2 or 4 weeks of UAC stimulation (n = 8). Similar to our previous study [8], there were no differences in the histomorphology or molecular properties of the samples from the left and right sides. Three TMJs from the right side of each group were prepared for histochemical, immunohistochemical, TUNEL and Safranin O staining (n = 3). Six right or left TMJs from each group were divided into three separate samples (n = 3) and were used for mRNA analysis using real-time PCR assays. The other seven left joints from each group were divided into three separate samples (n = 3) to assay protein levels using Western blotting.

For morphologic analysis, a tissue block containing the right TMJ was dissected out. The histological samples were fixed in 4% paraformaldehyde for 1 day and decalcified in 10% EDTA for 35 days. After dehydration, the tissue blocks were embedded in paraffin. A rotary microtome (Leica RM2135, Leica Microsystems, Nussloch GmbH, Germany) was used to make serial 4-μm-thick sections through the TMJ in the sagittal plane. The sections were mounted onto poly-L-lysine-coated glass slides. Hematoxylin&eosin and Safranin O staining was performed to determine changes in morphology and proteoglycans, respectively. TUNEL staining was performed to detect apoptosis in samples from every group. To ensure a reliable comparison between the specimens from different groups, the central sagittal sections of each joint were selected.

For Western blot and real-time PCR analysis, the cartilage was carefully isolated to exclude bone tissue and preserved at -80°C for protein or mRNA preparation. To ensure that the technical parameters of each stain were identical and that the results were comparable between groups, the sections from each group for one type of staining were stained at the same time.

### Histochemical and Immunohistochemical staining

**Fig 1:**
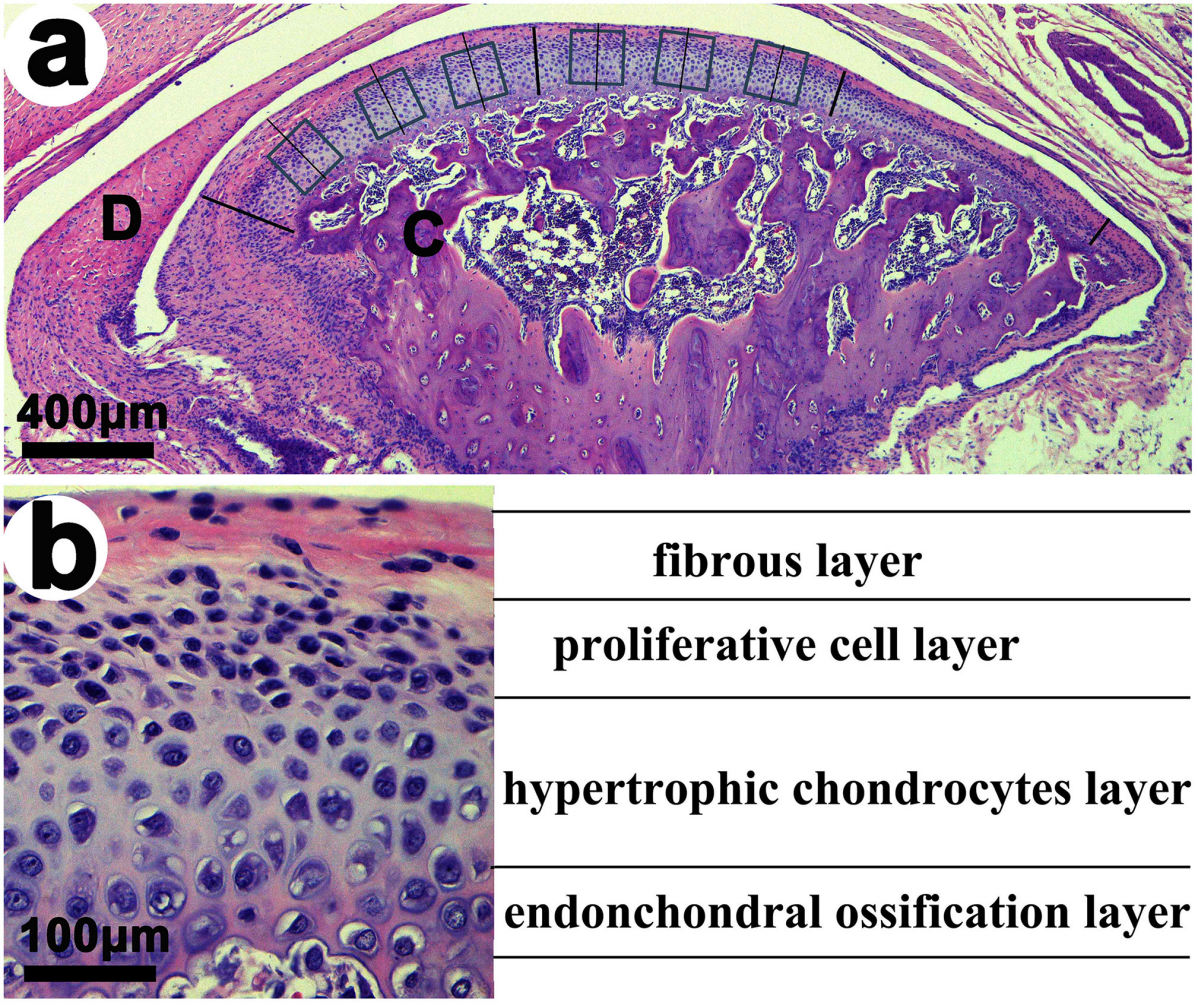
Sagittal central section of the TMJ in the control group stained with hematoxylin & eosin. **(a).** Six squares denoting the areas on slides where immunohistochemical and TUNEL staining were performed. D, articular disc; C, mandibular condyle. (**b).** Sagittal central section of the TMJ condylar cartilage showing the four cellular layers.

Hematoxylin&eosin (H&E) and immunohistochemical (IHC) staining was performed, as previously reported [8]. Immunohistochemistry staining with an anti-TNF primary antibody (diluted 1:50, sc8436, Santa Cruz) was performed using an avidin-biotin complex (ABC) IHC staining protocol. Negative controls were stained with non-immune serum instead of the primary antibody. Sections were mounted with balsam after being dehydrated in serial alcohol solutions. H&E, Safranin O and IHC stained sections were analyzed under a light microscope (Leica DM 2500, Wetzlar, Germany) and images were acquired using a Leica DFC490 system, as reported previously [7]. Briefly, Photoshop CS3 software was used to divide the surface of the cartilage into three parts consisting of the anterior, center, and posterior thirds between the anterior and posterior attachment positions of the condyle to the disk. Three squares (300 pixels × 300 pixels) covering all hypertrophic layers were applied at the quartering points of the posterior and middle thirds of the condylar cartilage (Fig 1a). The lengths of three short lines crossing through the hypertrophic layer were measured in each portion and averaged to determine the thickness of the hypertrophic layer in each third. The percentage of stain-positive cells in the selected squares was detected with an image analyzing system (Leica Qwin.Plus, Leica Microsystem Imaging, Cambridge), and the average value of six squares was calculated for statistical analysis.

### TUNEL analysis

Apoptotic chondrocytes in the condyles were assessed by TUNEL analysis with an *in situ* Cell Death Fluorescein Detection Kit (Roche) following the manufacturer’s protocol. Briefly, after deparaffinization and rehydration, sections were incubated with proteinase K (F. Hoffmann-La Roche Ltd., Diagnostics Division) for 50 min at 37°C. After rinsing with PBS, the sections were incubated with the reaction mixture solution for 120 min at 37°C. The slides were mounted and analyzed under a fluorescence microscope (BX-60, Olympus, Tokyo, Japan) with 450–500 nm excitation filters and an emission of 515–565 nm. Positive and negative controls were incubated with 0.01 mg/ml bovine pancreatic DNase I (Boehringer Mannheim GmbH) or labeling solution, respectively. Immunofluorescent positive cells in cartilage were counted and compared between groups.

### Chondrocyte isolation, culture and treatment with TNF and TNF inhibitor

Primary chondrocytes were isolated from the 6-week-old rat mandibular condylar cartilage through enzymatic digestion for 20 min with 0.25% parenzyme, followed by 2 h with 0.2% type II collagenase in RPMI 1640. The isolated cells were collected by brief centrifugation and were then resuspended in RPMI 1640 supplemented with 10% (v/v) FBS, 50 mg/ml streptomycin and 50 units/ml penicillin (Gibco). The cells were plated in culture dishes at a density of 5×10^7^ cells/cm^2^. The medium was replaced every 2 days, and the cells reached confluence after approximately 5 days in culture. The isolated chondrocytes were treated with 10, 50 or 100 ng/ml of TNF for 24 h. Chondrocytes in another group were treated with TNF 100 ng/ml and TNF inhibitor 10 ug/ml together for 24 h and were then isolated for further examination.

### Isolation of mitochondria

Mitochondria were isolated from chondrocytes from the TMJ condylar cartilage, which was carefully separated from bone tissue according to previously described procedures [42]. Mitochondria isolation kits for cultured cells and tissues (Beyotime, China) were used to isolate mitochondrial and cytosolic fractions from chondrocytes and condylar cartilage according to the manufacturer’s protocol. Briefly, chondrocytes (5x10^7^ cells) or condyle cartilage (100 g) samples were collected by trypsinization and centrifuged at 600 g for 5 min at 4°C. Then, the samples were incubated with 800 μL of mitochondria isolation reagent and homogenized on ice in a Dounce homogenizer. Unlysed cells and large debris were pelleted by centrifugation at 700 g for 10 minutes at 4°C. The supernatant was further centrifuged at 12,000 g for 15 minutes at 4°C. The supernatant (cytosolic fraction) was collected, and the pellet (mitochondrial fraction) was lysed in 100 μL of mitochondria splitting reagent to obtain mitochondrial fractions.

### Western blot detection of mitochondrial cytochrome c release

Western blot analysis was used to detect the cytochrome c content in the mitochondria and cytosol of chondrocytes in TMJ condylar cartilage, which was carefully removed to exclude bone tissue, and of cultured chondrocytes. The protein concentrations were quantified using the bicinchoninic acid (BCA) method with a protein assay kit (Pierce). The protein aliquots (30 μg) were separated using 12% SDS/polyacrylamide gel electrophoresis (PAGE) and transferred to polyvinylidene fluoride membranes (Millipore). After blocking in 5% non-fat dried milk in Tris-buffered saline (TBS) with 0.1% Tween-20 (TBST), pH 7.4, for 60 min at room temperature, the membranes were incubated overnight with purified rabbit anti-cytochrome c monoclonal antibodies (diluted 1:2000, Abcam) at 4°C. After washing with TBST, the membranes were incubated with secondary horseradish peroxidase (HRP)-conjugated IgG at room temperature for 1 h. The blots were then visualized by enhanced chemiluminescence with ECL reagents (Pierce).

### Measurement of caspase-8 and caspase-9 activity

The activity of caspase-8 and caspase-9 were determined using fluorogenic assay kits (Beyotime, China). The chondrocytes and TMJ condylar cartilage, which were carefully removed to exclude bone tissue, were lysed, and the extracts (30 mg protein) were incubated for 2 h with 2 mM of the specific fluorogenic peptide substrates Ac-IETD-pNA (acetyl-Ile-Glu-Thr-Asp p-nitroanilide) for active caspase-8 and Ac-LEHD-pNA (acetyl-Leu-Glu-His-Asp p-nitroanilide) for active caspase-9. The samples were read in a microplate spectrophotometer (Exl800, Biotek) with a 405-nm emission filter.

### RNA extraction and real-time PCR

The condylar cartilage samples from every group were homogenized and the total RNA was extracted using the Tripure Isolation Reagent (Roche). Primers for the targeted genes were designed as shown in Table 1. All genes were analyzed using CFX 96 real-time PCR (Bio-rad). Each target gene was analyzed three times relative to GADPH and the mean values were derived using the formula 2^-ΔΔCt^. The results were calculated as the relative fold increase/decrease of the target gene compared to the 2-week Control group.

**Table 1:** Gene primers.

Genes	Forward primer	Reverse primer
TNF	AACTCGAGTGACAAGCCCGTAG	GTACCACCAGTTGGTTGTCTTTGA
Caspase3	GCAGCAGCCTCAAATTGTTGAC	TGCTCCGGCTCAAACCATC
Caspase8	TGGTATATCCAGTCACTTTGCCAGA	CTCACATCATAGTTCACGCCAGTC
Caspase9	CTGAGCCAGATGCTGTCCCATA	GACACCATCCAAGGTCTCGATGTA
IL-1β	CTTCGTTAAATGACCTGCAGCTTG	AGGTCGGTCTCACTACCTGTGATG
IL-6	CCACTTCACAAGTCGGAGGCTTA	GTGCATCATCGCTGTTCATACAATC
GAPDH	GGCACAGTCAAGGCTGAGAATG	ATGGTGGTGAAGACGCCAGTA

#### Flow cytometry

After 24 h of treatment, 1×10^6^ cells were harvested and placed in EP tubes. The cells were rinsed with 0.1 mmol/L PBS (pH = 7.4) and collected by brief centrifugation. After that, the cells were resuspended in 190 μL of binding buffer, mixed with 5 μL of Annexin V-FITC, and then kept in darkness for 10 min at room temperature. Then, 10 μL of propidium iodide (PI) was added and the samples were kept in darkness for 10 min at room temperature. Approximately 300 μL of binding buffer was added, and the samples were analyzed using flow cytometry within 1 h. The blank control was the cells of the control group without Annexin V-FITC and PI. The Annexin V-FITC single-labeled control consisted of cells labeled with Annexin V-FITC. The PI single-labeled control consisted of cells labeled with PI.

#### Statistical analysis

Statistical analysis was accomplished using the SPSS 21.0 software. All data acquisition and analysis were completed blindly. Student’s *t*-test was used to compare two groups. For multiple comparisons of three or more groups, one-way analysis of variance with Tukey’s *post hoc* test was used. The results were expressed as the mean values with 95% confidence intervals (95% CI). *p* values less than 0.05 were considered to be statistically significant in all cases, and each experiment was performed in triplicate.

## Results

### 1. UAC induced degenerative changes in the mandibular condyle cartilage

**Fig 2:**
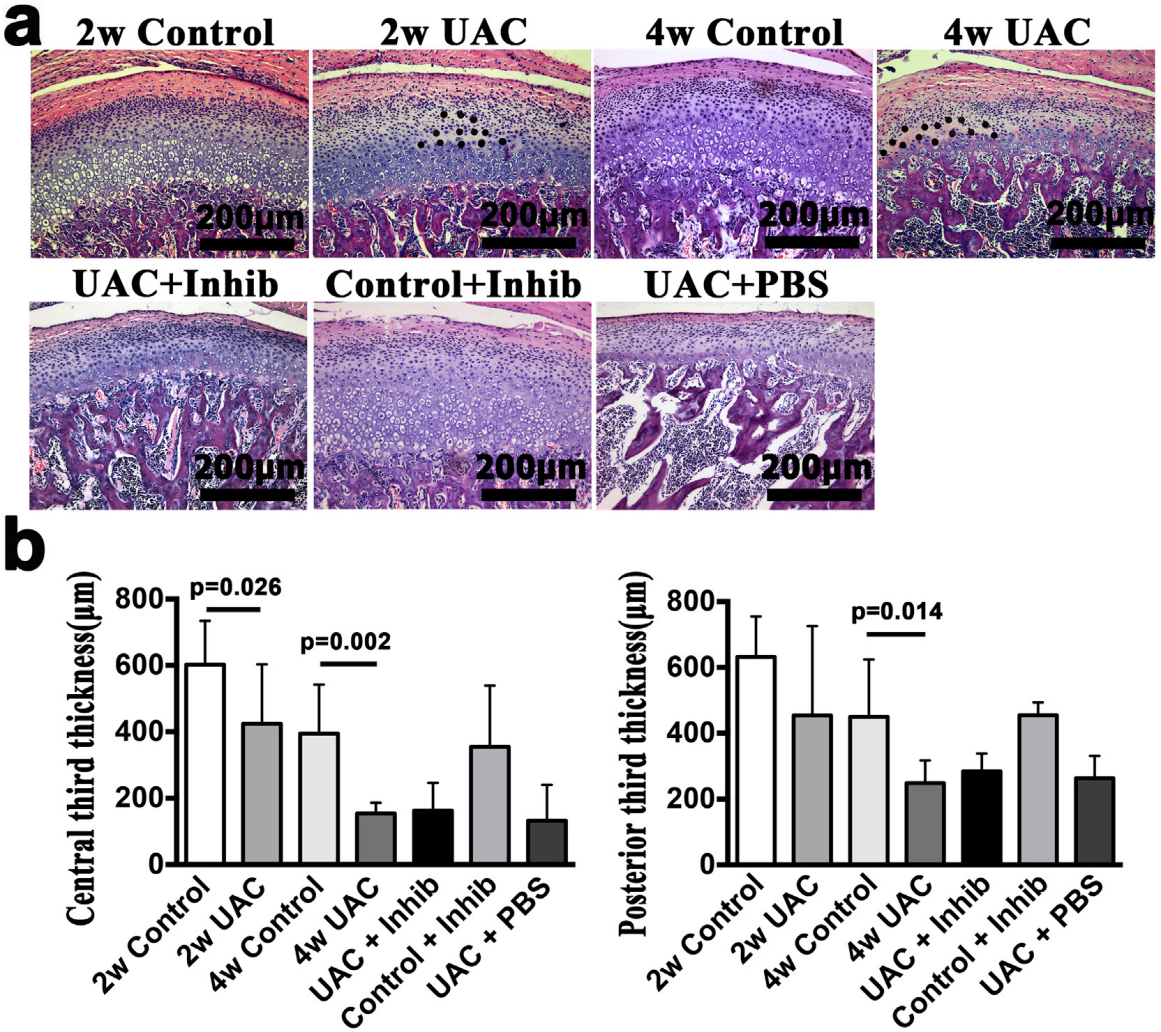
Condylar cartilage histomorphology staining. **(a).** A comparison of the TMJ condylar cartilage histomorphology between groups: 2-week Control (2w Control), 2-week UAC (2w UAC), 4-week Control (4w Control), 4-week UAC (4w UAC), UAC+Inhibitor (UAC+Inhib), Control+Inhibitor (Control+Inhib) and UAC+PBS (UAC+PBS). Areas in 2w UAC and 4w UAC where cells have died are indicated by points. **(b).** Comparison of cartilage thickness in the central and posterior thirds. The data are expressed as the means and 95% CIs. n = 3.

**Fig 3:**
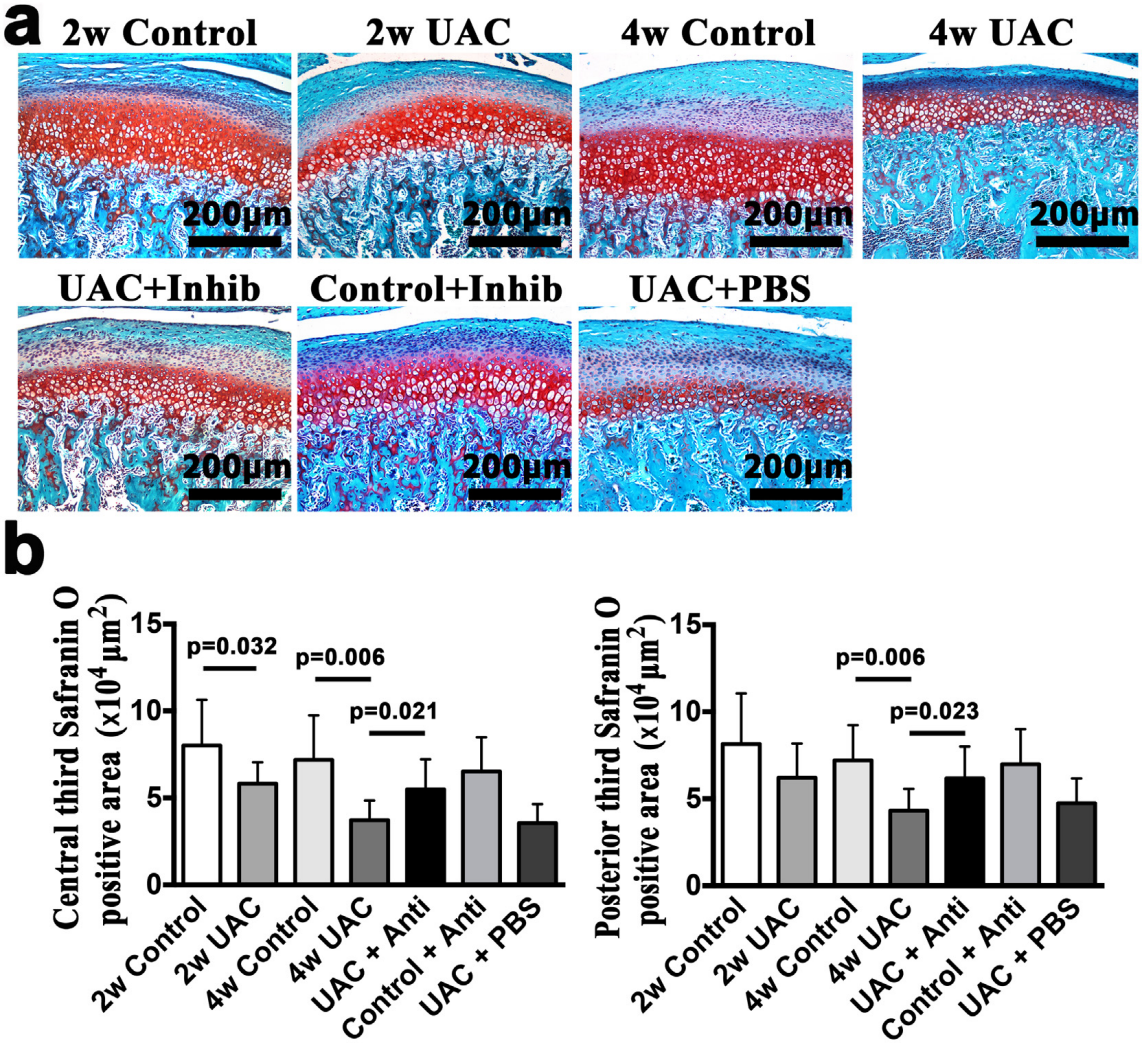
Condylar cartilage Safranin O staining. **(a).** Safranin O staining between groups: 2-week Control (2w Control), 2-week UAC (2w UAC), 4-week Control (4w Control), 4-week UAC (4w UAC), UAC+Inhibitor (UAC+Inhib), Control+Inhibitor (Control+Inhib) and UAC+PBS (UAC+PBS). **(b).** Comparison of Safranin O staining positive areas in the central and posterior thirds. The data are expressed as the means and 95% CIs. n = 3.

**Fig 4:**
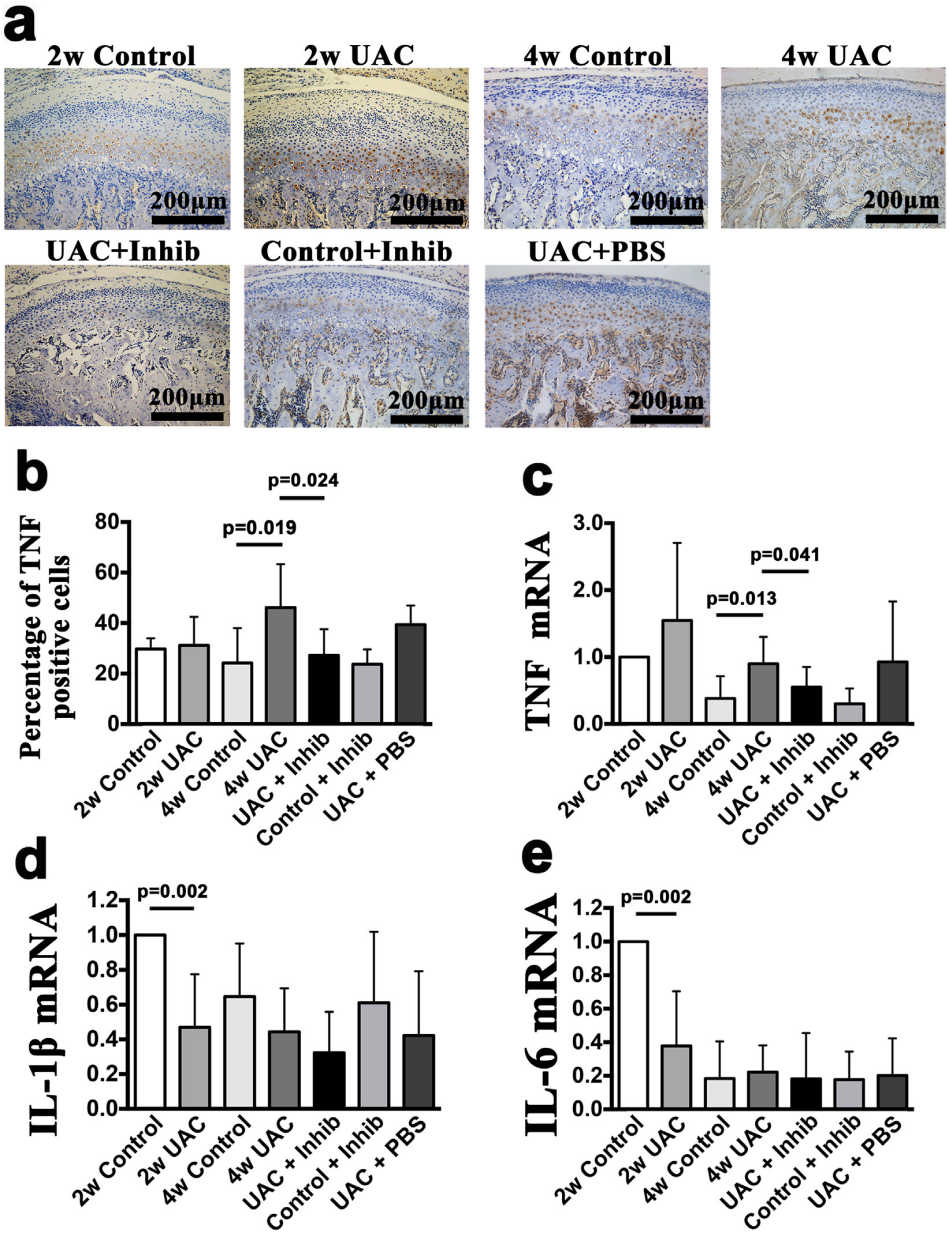
Detection of inflammatory factors. Comparison of the immunohistochemical staining of TNF **(a)**, the percentage of TNF positive cells **(b)** and the mRNA expression levels of TNF **(c)**, IL-1β **(d)** and IL-6 **(e)** in the mandibular condylar cartilage between the 2-week Control (2w Control), 2-week UAC (2w UAC), 4-week Control (4w Control), 4-week UAC (4w UAC), UAC+Inhibitor (UAC+Inhib), Control+Inhibitor (Control+Inhib) and UAC+PBS (UAC+PBS) groups. The data are expressed as the means and 95% CIs. n = 3.

The condylar cartilage in the control group typically included the fibrous, proliferative, hypertrophic and endochondral ossification layers [8] (Figs 1b and 2a). Safranin O staining showed that proteoglycans were evenly distributed (Fig 3a). IHC staining indicated that TNF-positive cells were located mainly in the hypertrophic layer (Fig 4a). UAC induced OA-like changes in the cartilage after 2 weeks, as previously reported [8, 43, 44]. These lesions were characterized by reduced numbers and size of chondrocytes in the cartilage, pyknotic nuclei, cellular disarrangement, condensed cytoplasm that did not fill the lacunae, and even cell-free areas (Fig 2a). The cartilage was thinner in the central and posterior thirds in the 2-week UAC and 4-week UAC groups than in the age-matched controls (central third: *p* = 0.026 for the 2-week group, *p* = 0.002 for the 4-week group; posterior third: *p* = 0.128 for the 2-week group, *p* = 0.014 for the 4-week group) (Fig 2b). The proteoglycan levels were lower at both time points (central third: *p* = 0.032 for the 2-week group, *p* = 0.006 for the 4-week group; posterior third: *p* = 0.077 for the 2-week group, *p* = 0.006 for the 4-week group) (Fig 3b). TNF-positive cells were observed not only in the hypertrophic layer, but also in the pre-hypertrophic and proliferative layers (Fig 4a). The percentage of TNF-positive cells and the TNF mRNA expression level were increased at 4 weeks (IHC: *p* = 0.019, real-time PCR: *p* = 0.013), but not at 2 weeks (IHC: *p* = 0.616, real-time PCR: *p* = 0.063) (Fig 4b and 4c). The mRNA expression level of IL-1β (*p* = 0.002) and IL-6 (*p* = 0.002) in the UAC group were down-regulated at 2 weeks and were identical to the control group at 4 weeks (Fig 4d and 4e). There were no signs of inflammatory cell infiltration in the degraded cartilage.

### 2. UAC induced TMJ chondrocyte apoptosis via both the mitochondrial pro-apoptotic and TNF receptor pathways

**Fig 5:**
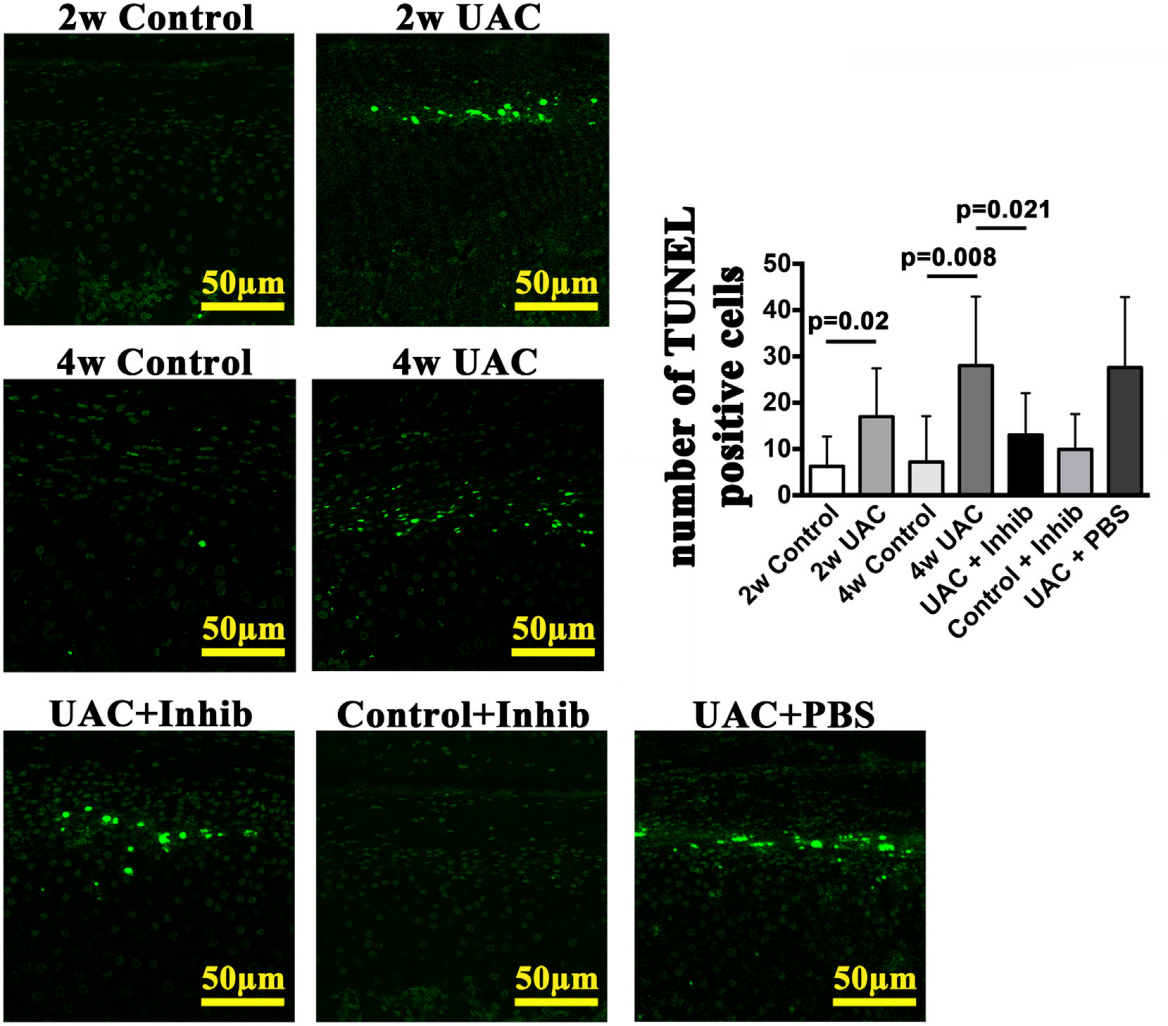
TUNEL staining. TUNEL staining images and between-group comparisons of the numbers of TUNEL-positive cells of the 2-week Control (2w Control), 2-week UAC (2w UAC), 4-week Control (4w Control), 4-week UAC (4w UAC), UAC+Inhibitor (UAC+Inhib), Control+Inhibitor (Control+Inhib) and UAC+PBS (UAC+PBS) groups. The data are expressed as the means and 95% CIs. n = 3.

**Fig 6:**
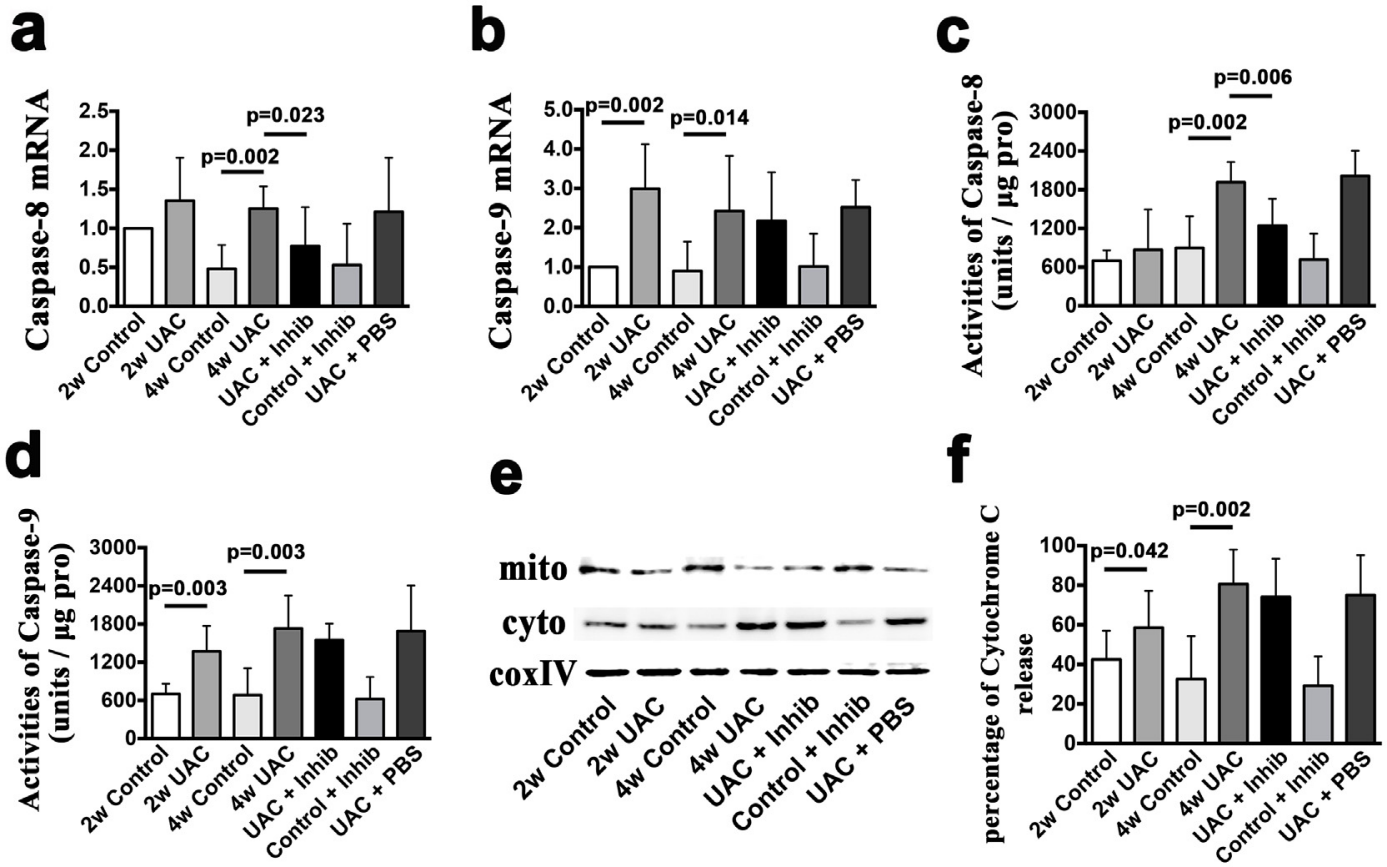
The mRNA and protein levels of apoptotic pathway components *in vivo*. A comparison of the apoptotic activity in the mandibular condylar cartilage between the 2-week Control (2w Control), 2-week UAC (2w UAC), 4-week Control (4w Control), 4-week UAC (4w UAC), UAC+Inhibitor (UAC+Inhib), Control+Inhibitor (Control+Inhib) and UAC+PBS (UAC+PBS) groups. The mRNA expression levels of caspase-8 **(a)** and caspase-9 (**b**). The activities of caspase-8 **(c)** and caspase-9 **(d)** units. Western blot for the release of cytochrome c from the mitochondria into the cytoplasm **(e)** and its quantification **(f)**. The data are expressed as the means and 95% CIs. n = 3.

In the TMJ cartilage of the control group, there were only a few scattered TUNEL-positive chondrocytes. However, in both the 2-week UAC and 4-week UAC groups, the numbers of TUNEL-positive cells were increased (*p* = 0.02 and *p* = 0.008, respectively). The TUNEL-positive cells were located mainly in the proliferative and hypertrophic layers of the condylar cartilage (Fig 5). The mRNA expression of caspase-9 was up-regulated at 2 weeks (*p* = 0.002 for the 2-week group; *p* = 0.014 for the 4-week group) (Fig 6b). However, the mRNA expression level of caspase-8 was not up-regulated until 4 weeks (*p* = 0.002) (Fig 6a). Furthermore, fluorogenic analysis revealed that the level of active caspase-9 units was increased at both 2 and 4 weeks (both *p* = 0.003) (Fig 6c and 6d), whereas the level of active caspase-8 units was not increased until 4 weeks (2-week group: *p* = 0.384, 4-week group: *p* = 0.002). The level of cytochrome c release from the mitochondria into the cytosol was also increased at both 2 and 4 weeks (*p* = 0.042 and *p* = 0.002, respectively) (Fig 6e and 6f).

### 3. Anti-TNF treatment partially reduced UAC-induced degenerative changes and chondrocyte apoptotic activity

Intra-articular injection of the TNF inhibitor partially reduced the loss of proteoglycans, as demonstrated by Safranin O staining (Fig 3a), and reduced the TNF expression level (TNF-positive cells: *p* = 0.024; TNF mRNA: *p* = 0.041) (Fig 4b and 4c) in the condylar cartilage. However, no effects on the reduced cartilage thickness were observed (Fig 2a and 2b). The mRNA expression levels of IL-1β and IL-6 were not affected (*p*>0.05) (Fig 4d and 4e). The TNF inhibitor also reduced the number of TUNEL-positive cells stimulated by UAC (*p* = 0.021) (Fig 5), the mRNA expression level of caspase-8 (*p* = 0.023), and the levels of active caspase-8 units (*p* = 0.006), but had no effect on the mRNA expression levels of caspase-9, the levels of active capsase-9, or the cytosolic levels of cytochrome c (Fig 6a–6f). The TNF inhibitor had no effect on the TMJ cartilage in the control group, and did not rescue the UAC-induced degradation of TMJ cartilage.

### 4. TNF stimulated chondrocyte death via the TNF receptor pathway

**Fig 7:**
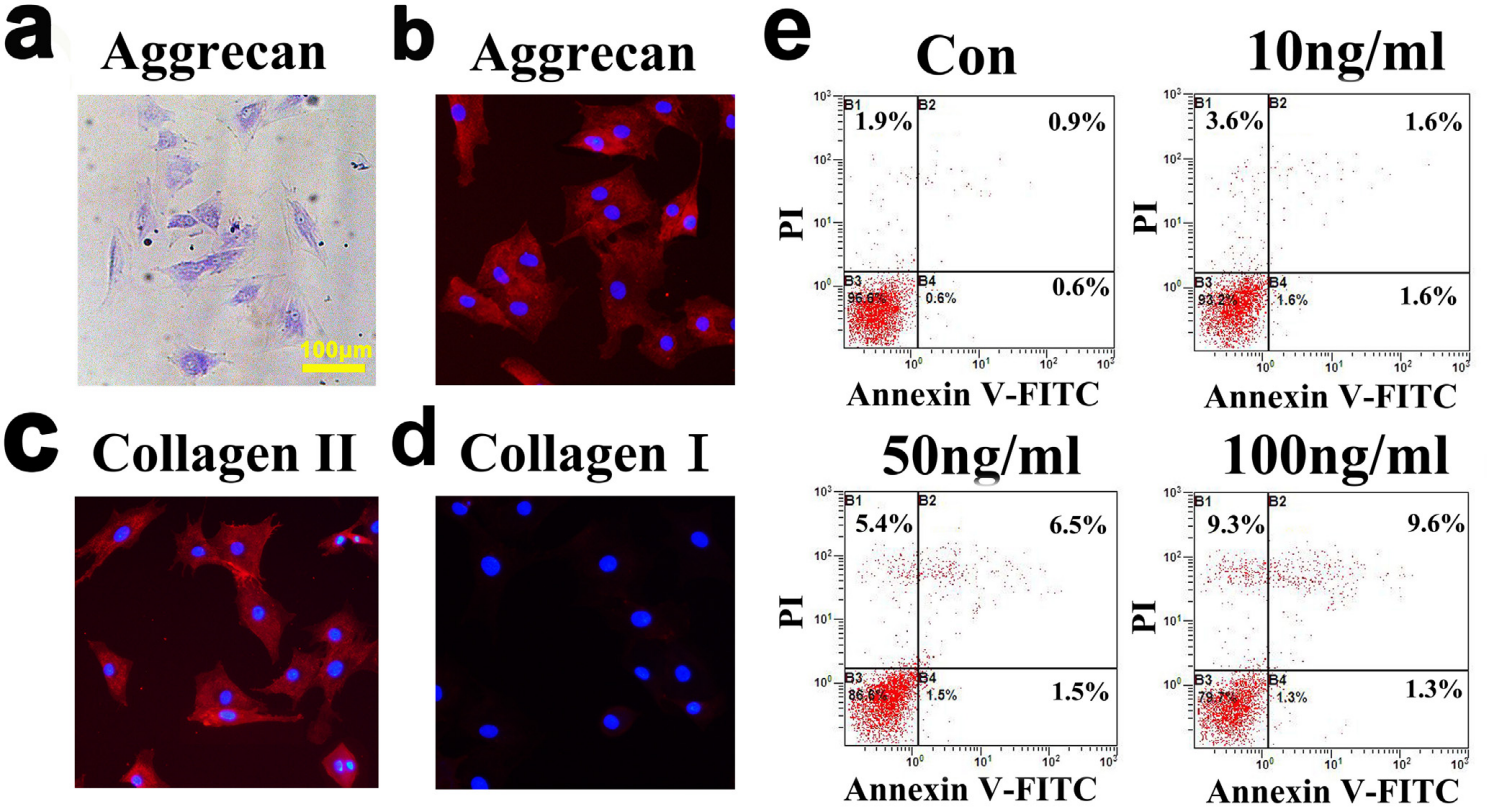
Cell identification and detection of apoptosis in cultured chondrocytes. **(a).** Chondrocytes identified by toluidine blue staining. **(b).** Aggrecan staining (Primary antibody: abcam, Ab36861. Secondary antibody: Zhongshan Co. Ltd, China, Cy3-conjugated goat anti-rabbit IgG). **(c).** Type II collagen dyeing (Primary antibody: Santa Cruz, sc7763. Secondary antibody: Zhongshan Co. Ltd, China, Cy3-conjugated donkey anti-goat IgG). **(d).** Type I collagen dyeing (Primary antibody: Abcam, ab90395. Secondary antibody: Zhongshan Co. Ltd, China, FITC-conjugated goat anti-mouse IgG). **(e).** Chondrocyte apoptosis detected by flow cytometry after stimulation with TNF at dosages of 10 ng/ml, 50 ng/ml or 100 ng/ml for 24 h.

**Fig 8:**
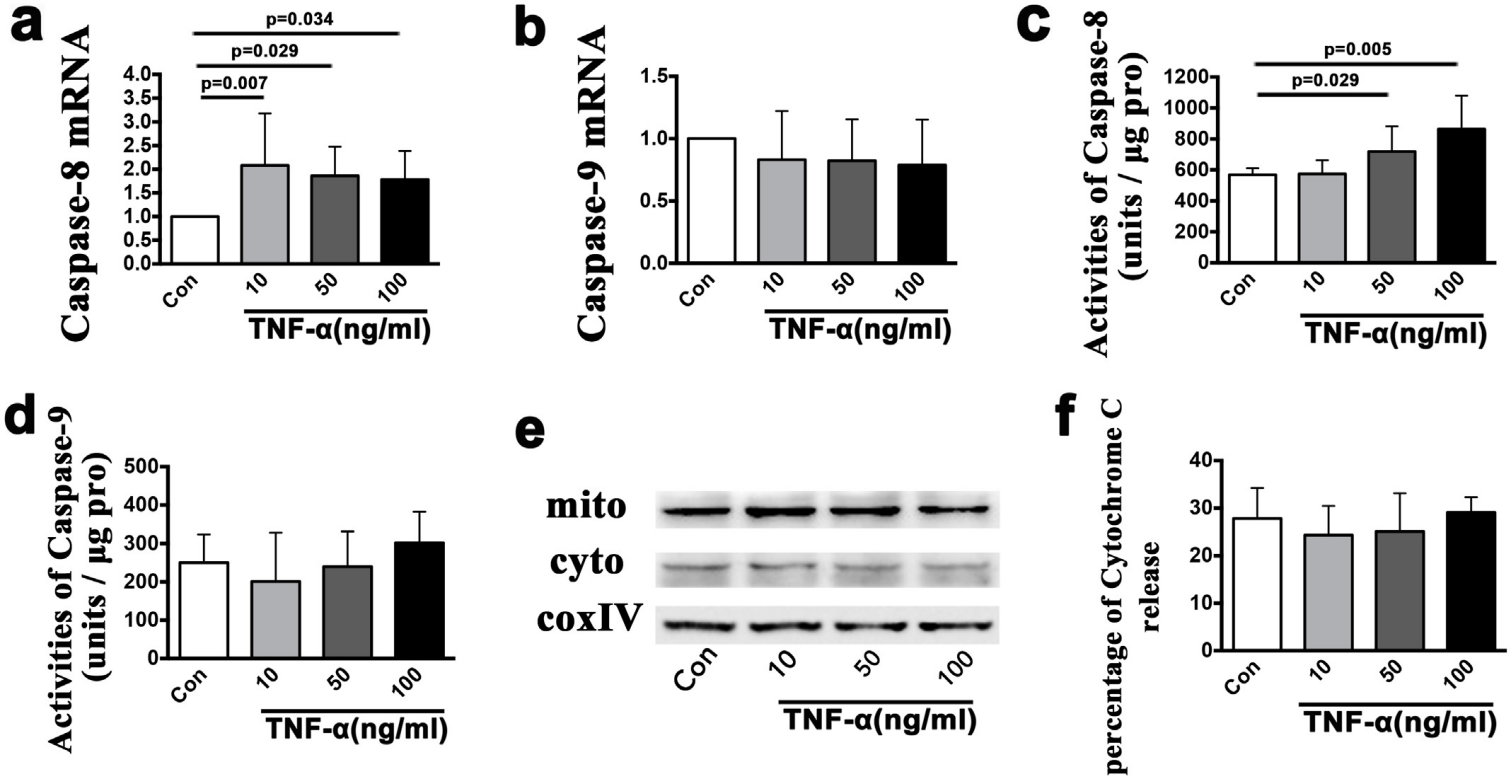
The mRNA and protein levels of apoptosis *in vitro*. A comparison of the mRNA expression levels of caspase-8 **(a)** and caspase-9 **(b)** in the cultured chondrocytes between groups with or without TNF (10 ng/ml, 50 ng/ml and 100 ng/ml) stimulation for 24 h. Comparison of the activities of caspase-8 **(c)** and caspase-9 **(d)** units between the groups with or without TNF (10 ng/ml, 50 ng/ml and 100 ng/ml) stimulation for 24 h. Western blot measuring the release of cytochrome c from the mitochondria to the cytoplasm **(e)** and comparisons between the groups with or without TNF (10 ng/ml, 50 ng/ml and 100 ng/ml) stimulation for 24 h **(f)**. The columns represent the mean values with 95% CIs. n = 3.

**Fig 9:**
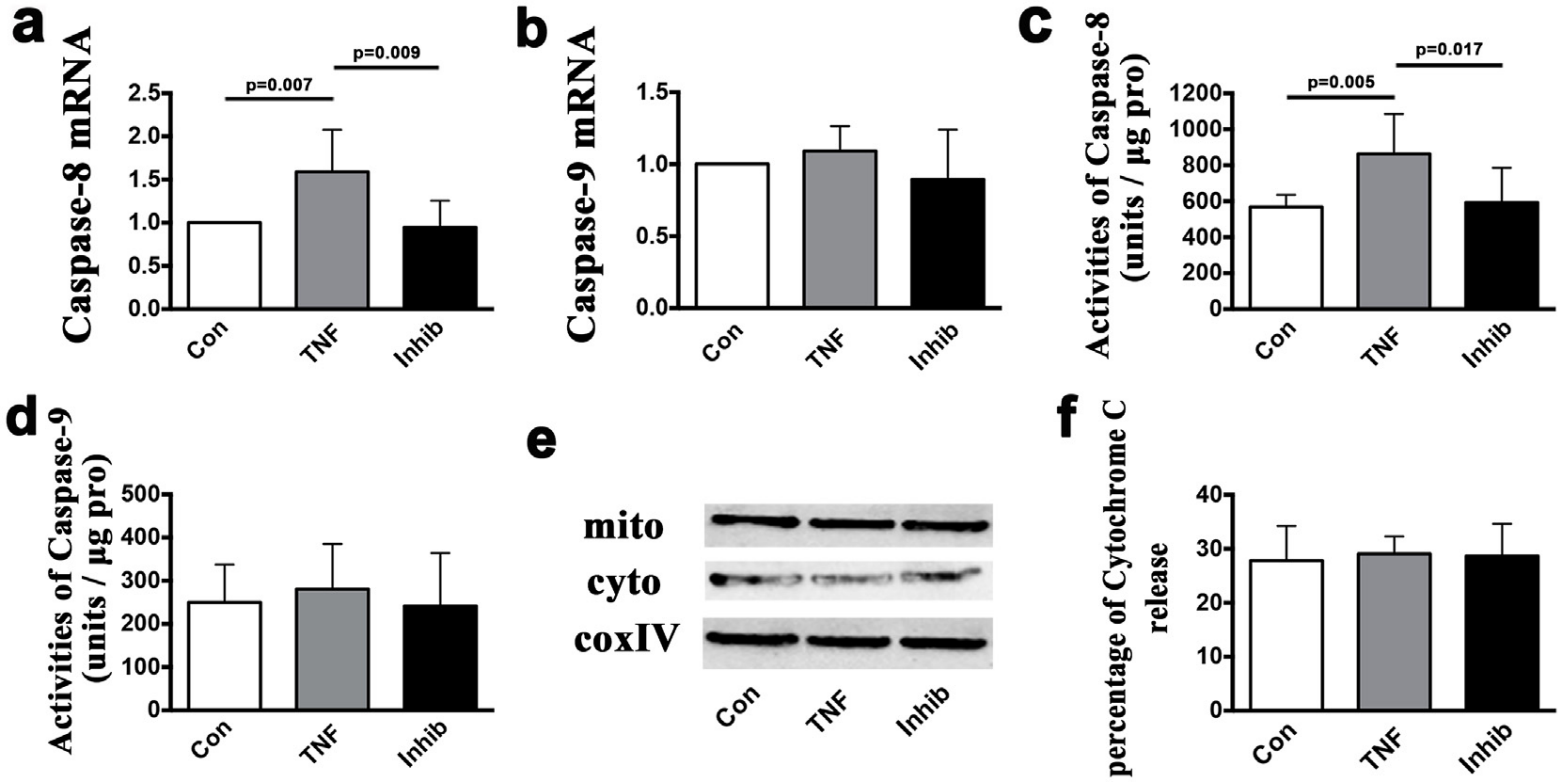
The mRNA and protein levels *in vitro* after treatment with TNF inhibitor. A comparison of the mRNA expression levels of caspase-8 **(a)** and caspase-9 **(b)** in the cultured chondrocytes stimulated for 24 h between the groups: control (Con), TNF 100 ng/ml (TNF) and TNF 100 ng/ml plus TNF inhibitor 10ug/ml (Inhib). Comparison of the activities of caspase-8 **(c)** and caspase-9 **(d)** units between the groups with stimulation for 24 h. Western blot measuring the release of cytochrome c from the mitochondria to the cytoplasm **(e)** and comparisons between the groups with stimulation for 24 h **(f)**. The columns represent the mean values with 95% CIs. n = 3.

The viability of isolated TMJ chondrocytes was determined using toluidine blue staining, aggrecan, type I collagen, and type II collagen staining (Fig 7a–7d). Almost all of the cells were toluidine blue-, aggrecan- and type II collagen-positive, but type I collagen staining was not observed. After exposure to 10, 50 or 100 ng/ml of TNF for 24 h, the percentage of FITC+/PI- viable apoptotic cells within the isolated TMJ chondrocytes increased from 0.6% without TNF stimulation to 1.6%, 1.5% and 1.3%, respectively, whereas the percentage of FITC+/PI+ non-viable apoptotic cells increased from 0.9% without TNF stimulation to 1.6%, 6.5% and 9.6%, respectively. Similar results were found in the percentage of FITC-/PI+ necrotic cells, which were 1.9% without TNF stimulation and 3.6%, 5.4% and 9.3% after stimulation with 10, 50 and 100 ng/ml of TNF, respectively (Fig 7e). The mRNA expression level of caspase-8 was significantly up-regulated after stimulation with all three concentrations of TNF (10 ng/ml: *p* = 0.007; 50 ng/ml: *p* = 0.029; 100 ng/ml: *p* = 0.034), though the mRNA expression levels of caspase-9 was not up-regulated (*p*>0.05) (Fig 8a and 8b). Fluorogenic analysis revealed that the levels of active caspase-8 increased after stimulation with 50 or 100 ng/ml of TNF (*p* = 0.029 and *p* = 0.005) (Fig 8c). However, neither the levels of active caspase-9 nor the cytosol cytochrome c levels were increased (Fig 8d, 8e and 8f). Addition of the TNF inhibitor (10 μg/ml) to TNF (100 ng/ml) treated chondrocytes for 24 h reversed the increased levels of caspase-8 mRNA expression (*p* = 0.009) and of active caspase-8 (*p* = 0.017) (Fig 9a and 9c), but had no effect on the mRNA expression levels of caspase-9, the levels of active capsase-9, or the cytosolic levels of cytochrome c (Fig 9b, 9d, 9e and 9f).

## Discussion

Temporomandibular joint disorder (TMD) is common and is induced by various pathologic conditions, including aberrant occlusion [45]. TMJ-OA is a severe form of TMD, and chondrocyte apoptosis is one of the main characteristics of the arthritic cartilage [9]. In this study, we found that UAC induced chondrocyte apoptosis as early as 2 weeks after initiation. Degeneration in the TMJ cartilage was associated with up-regulation of expression of the pro-inflammatory cytokine, TNF, which was observed at 4 weeks. The expression levels of caspase-9 and cytochrome c release both increased by 2 weeks, while the caspase-8 expression levels did not increase until 4 weeks. These findings indicate that the traumatic stimulation of UAC induced chondrocyte apoptosis via the mitochondrial pro-apoptotic pathway, which was followed by TNF-mediated apoptosis in the chondrocytes. This agrees with the findings of Dechao Kong et al. [46], who reported that static mechanical stress induces apoptosis first through activation of mitochondrion-dependent, caspase-mediated signaling pathways. Our *in vitro* data agree with this finding, demonstrating that caspase-8 is involved in the TNF-stimulated apoptosis of chondrocytes without involvement of either caspase-9 or cytochrome c at all three dosages. At the highest dose of TNF (100 ng/ml) used to treat chondrocytes, the TNF inhibitor (10ug/ml) prevented the caspase-8 increase without affecting either caspase-9 or cytochrome c. This compliments the *in vivo* data showing that injection of the TNF inhibitor down-regulated the levels of caspase-8, but not caspase-9 or the release of cytochrome c. Moreover, this treatment effect of the TNF inhibitor in the presence of TNF confirmed that this anti-TNF monoclonal antibody works as a neutralizing antibody.

Some studies have suggested that the mandibular condylar cartilage plays a significant role during mandibular development and has been referred to as secondary cartilage [47], which is different from the primary cartilage seen in knee joints. Others have reported that there is a unique fibrous layer on the superficial zone of the cartilage contained some fibroblasts that could synthesize type I collagen [48], and that this layer significantly reduces the deformation of condylar cartilage under compression and the friction force on its surface [49]. However, all of the cells we currently isolated from TMJ condyles were positive for toluidine blue, aggrecan and type II collagen staining, but not for type I collagen. This agrees with the results of Klinge [50] and Landesberg et al [51], who found that the largest cells in condyles were hypertrophic chondrocytes, while the smallest cells were fibroblast-like chondrocytes. There were only a few fibroblast-like chondrocytes, which could synthesize type I collagen [50] and these were located in the anterior third upper zones of the mandibular condylar cartilage [52].

Intra-articular injection of a TNF monoclonal antibody model has been reported to control local inflammation and inhibit the degradation of the cartilage matrix in an animal model of TMJ OA [39], and also to control the debilitating pain, stiffness and function observed in OA patients [53, 54]. However, reports from other clinical and animal studies do not support these findings. For example, treatment with adalimumab (an anti-TNF antibody) for 3 months did not significantly improve the signs and symptoms of patients with erosive or inflammatory osteoarthritis, except for rare relief of pain [55]. As reviewed by Fisher and Keat [56], the effect of intra-articular treatment with anti-TNF drugs is not supported by the current literature, although the available data seem encouraging. Our data demonstrate that a TNF inhibitor rescued those chondrocytes that were most likely to undergo apoptosis via the TNF receptor pathway, though it is possible that some chondrocyte apoptosis was induced via the intrinsic mitochondrial pathway initiated by the dental biomechanical stimulation we induced. Inhibiting TNF only partially reversed the OA lesions, prevented loss of the Safranin O-stained tissue in the cartilage matrix, or suppressed the increase in TUNEL-positive cells, but did not increase the cartilage thickness. The extremely high concentration of 10 μg/ml of the anti-TNF monoclonal antibody showed appropriate pharmacokinetic properties, and resulted in no toxicity in animals [56]. Although a number of pharmacokinetic issues should be kept in mind when interpreting the current results, including the effectiveness of different anti-TNF drugs, the times and frequency of injection, and the drug concentrations [57], the present results suggest that this therapeutic approach has at least partial efficacy in rescuing induced OA. Our recently published report showing that removal of UAC at 3 weeks successfully rescued the induced TMJ OA lesions in mice by the end of 7 weeks [58] indicate that reversal of mal-occlusion is an important component of any therapeutic approach.

TNF plays a pivotal role in induction of chondrocyte apoptosis and cartilage degeneration in OA [35] and has been reported to elicit the expression of other pro-inflammatory cytokines, such as IL-1β [37]. In contrast to other studies that used chondrocytes from OA patients and therefore provided data from a later stage of OA, our UAC model created an early stage of OA that allowed us to detect the earliest degenerative changes in OA. We did not detect the up-regulation of IL-1β or IL-6 during this 4-week study period and indeed found a temporary decrease in IL-1β and IL-6 expression, suggesting that these pro-inflammatory cytokines are less involved in the initial OA lesions than in the later OA lesions described in the literature [59].

In summary, we report that UAC stimulation induced apoptosis of chondrocytes in osteoarthritic cartilage, which was initiated by the mitochondrial pro-apoptotic signaling pathway and was secondarily promoted by increased expression of TNF and the extrinsic apoptotic pathway. Our results demonstrate limited efficacy of injecting a TNF inhibitor to prevent chondrocyte apoptosis in OA and the progression of OA lesions and our recently published findings indicate that removal of the aberrant biomechanical stimulation is pivotal for rescue of the OA lesions.

## Supporting Information

S1 Appendix The ARRIVE checklist for reporting animal studies. (PDF)
